# Burkitt's lymphoma: The Rosetta Stone deciphering Epstein-Barr virus biology

**DOI:** 10.1016/j.semcancer.2009.07.004

**Published:** 2009-12

**Authors:** Martin Rowe, Gemma L. Kelly, Andrew I. Bell, Alan B. Rickinson

**Affiliations:** Institute for Cancer Studies, School of Cancer Sciences, College of Medical and Dental Sciences, University of Birmingham, Edgbaston, Birmingham, B15 2TT, UK

**Keywords:** Epstein-Barr virus, Burkitt's lymphoma, Cancer pathogenesis, c-myc, Apoptosis

## Abstract

Epstein-Barr virus was originally identified in the tumour cells of a Burkitt's lymphoma, and was the first virus to be associated with the pathogenesis of a human cancer. Studies on the relationship of EBV with Burkitt's lymphoma have revealed important general principles that are relevant to other virus-associated cancers. In addition, the impact of such studies on the knowledge of EBV biology has been enormous. Here, we review some of the key historical observations arising from studies on Burkitt's lymphoma that have informed our understanding of EBV, and we summarise the current hypotheses regarding the role of EBV in the pathogenesis of Burkitt's lymphoma.

## 1 Introduction

Having characterised a childhood lymphoma that is endemic in geographically restricted areas of equatorial Africa, Denis Burkitt originally hypothesized that this tumour might be caused by an insect-borne vector [1,2]. That hypothesis seemed to be borne out by the subsequent discovery, by Epstein and co-workers, of a novel herpesvirus in a tumour cell line established from a Burkitt's lymphoma (BL) patient [3]. There is now a substantial body of evidence implicating a role for this virus, the Epstein-Barr virus (EBV), in the pathogenesis of most cases of BL (reviewed in [4]). EBV is not, however, an insect-borne agent. That role in BL belongs to *Plasmodium falciparum* malaria, which is transmitted by mosquitoes [5]. Furthermore, the key event in the pathogenesis of BL is now known to be the acquisition of a chromosomal translocation, involving the immunoglobulin gene loci on chromosomes 14, 22 or 2 and the c-myc oncogene on chromosome 8. This results in deregulated expression of the c-myc protein, and is a characteristic of all BL tumours including the minority that are not EBV-associated [6–8]. Through mechanisms that are only partially understood, malaria and EBV together exert effects on the human host to increase the likelihood of this genetic accident, and to synergize with the deregulated *c-myc* to enhance the survival and proliferative capacity of the tumour.

It has been suggested previously [9] that EBV is a metaphorical Rosetta Stone for understanding the role of viruses in immunopathological disorders and human carcinogenesis. To a large extent, it is true that the association of EBV with malignant diseases has become paradigmatic for viral oncology. Conversely, it is also the case that the study of BL has yielded many clues to the biology of EBV. In this review we shall highlight some of the observations that have profoundly enhanced the wider understanding of the interaction of EBV with normal and malignant cells.

## 2 EBV latent gene expression

Infection of resting primary B cells in culture leads to the establishment of growth-transformed lymphoblastoid cell lines (LCLs) [10,11]. This potent growth-transforming property of EBV, together with seroepidemiological data [12,13] and the demonstration of EBV DNA in the tumours of nearly all African BL patients [14–16], seemingly provided incontrovertible evidence for a role of EBV in the pathogenesis of BL. A critical assumption was made, however, that LCLs were good experimental models for the uncontrolled proliferation of BL tumours. As methods became available to analyse EBV gene expression in more detail, it was unexpectedly found that BL tumours express a much more restricted pattern of viral genes than do LCLs established from the normal B cells of the same patients [17–19]. In fact, EBV-transformed LCLs are a better model for B cell lymphoproliferations arising in immunosuppressed patients [20–22], which underlines the different etiologies of two EBV-associated tumours of B cell origin. The observation, that EBV can establish different latent states (i.e. infections not leading to replication of new virus particles) in normal and malignant B cells [18,23,24], had repercussions for understanding the role of EBV not only in BL, but also in other EBV-associated malignancies and in normal persistence in healthy infected individuals (*see below*).

### 2.1 Latency I and Latency III

More than 20 years after the first realization that EBV gene expression in BL cells differs from that in EBV-transformed normal LCLs, details are still emerging with the discovery of new forms of latency [25,26], new latent genes [27], and several virally encoded micro-RNAs [28–31].

**Fig. 1:**
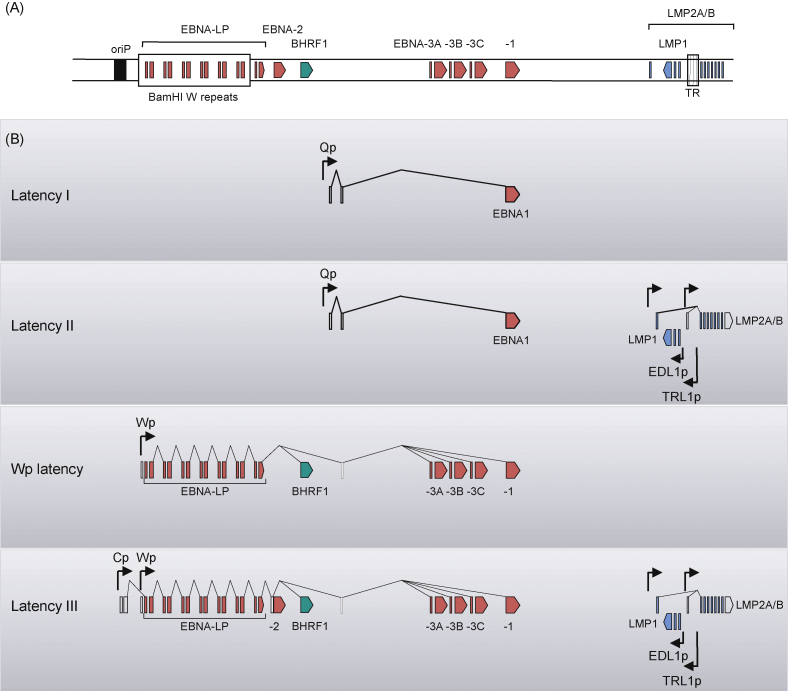
Patterns of EBV latent protein expression in different forms of viral latency. The top panel (A) is a schematic illustration of the EBV genome, showing the location of coding exons for the nuclear antigens EBNA1, EBNA2, EBNA3A, EBNA3B, EBNA3C and EBNA-LP; the viral bcl-2 homologue, BHRF1; and the three latent membrane proteins LMP1, LMP2A and LMP2B. Also shown are the latent origin of replication (oriP), the BamHI W internal repeat region and the fusion of the terminal repeats (TR). Shown below (B) are the structures of viral mRNA transcripts expressed in different forms of latency. Promoters are identified by arrowheads, coding exons are coloured and non-coding exons are unshaded. Latency I, seen in most endemic BL tumours, is characterised by the restricted expression of a single latent antigen EBNA1 transcribed from Qp. The Latency II form of infection seen in EBV-positive Hodgkin's lymphoma and NPC tumour cells is characterised by expression of the LMP proteins in addition to Qp-initiated EBNA1. The classical growth-transforming Latency III infection, displayed by *in vitro*-transformed LCLs, is characterised by expression of all ten latent cycle proteins; the different EBNA mRNAs are generated by alternative splicing of long primary transcripts initiated either from the tandemly repeated Wp promoter or the upstream Cp promoter, while separate promoters in the BamHI N region transcribe the LMPs. BHRF1, which has recently been described as a tenth latent antigen, is also expressed from Wp-initiated transcripts in Latency III. A fourth type of infection, termed Wp-restricted latency, is characterised by expression of EBNA1, EBNA3A, EBNA3B, EBNA3C, EBNA-LP and BHRF1 in the absence of EBNA2 and the LMPs, and is seen in a subset of endemic BL tumours which carry EBV genomes deleted for the EBNA2 gene.

It is now known that EBV transformed LCLs express at least 6 nuclear antigens (EBNA1, EBNA2, EBNA3A, EBNA3B, EBNA3C and EBNA-LP) and three membrane proteins (LMP1, LMP2A and LMP2B); in contrast, the majority of BL tumours express only one virally encoded protein, EBNA1. These two patterns of gene expression are often referred to as Latency III and Latency I, respectively [24], and they are achieved by the use of different promoters to generate alternative primary transcripts from which the different EBNA mRNAs are spliced (Fig. 1). In Latency I BL lines, EBNA1 transcripts are driven by the Qp promoter in the BamHI Q region of the genome [32,33], whereas in Latency III LCLs the EBNA transcripts are driven by either of two upstream promoters, Cp or Wp, in the BamHI C or W regions of the genome [34–37]. As we shall discuss later, expression of the BHRF1 protein (a Bcl-2 homologue with anti-apoptotic properties) is also associated with Latency III type infection, but is not expressed in Latency I [27].

### 2.2 Latency II

An intermediate pattern of viral gene expression was originally identified in undifferentiated nasopharyngeal carcinoma (NPC) [38–40], and subsequently in EBV-associated gastric carcinomas [41,42], Hodgkin's lymphomas [43,44], and T cell lymphomas [45]. In this Latency II type of infection, transcripts for the LMP1 and LMP2A/B proteins are expressed in addition to Qp-driven EBNA1, and none of the other EBNAs is expressed (Fig. 1). It should be noted that Latency II type infection defines a spectrum from EBNA1 expression with low levels of LMP2, typical of gastric carcinomas, to EBNA1 expression with high levels of both LMP1 and LMP2 that is typical of Hodgkin's lymphoma. Whilst B cells have the potential to support any of these three types of latent infection, non-B cells generally display either a Latency I or Latency II type of infection. This is due at least in part to the fact that activation of the Wp/Cp promoters necessary for Latency III is regulated by a B cell-specific transcription factor, BSAP (alternatively called PAX-5) [46].

In growth-transformed B cells displaying Latency III type infection, LMP1 and LMP2A/B expression are dependent upon expression of EBNA2 [47–49], whereas in epithelial cells supporting a Latency II type infection, these membrane proteins are expressed independently of EBNA2. Despite extensive study, the control of the LMP genes in different latent states is not well understood. One contributory mechanism is the use of alternative promoters. In B cells, expression of LMP1 and LMP2B in Latency III type infection is mediated by a bi-directional promoter that is proximal to the LMP1 gene; this promoter, known as ED-L1p, is activated by an EBNA2-dependent mechanism involving the transcription factors RBP-Jκ, PU.1, POU, and AP-2 [47,50,51]. In epithelial cells a distal LMP1 promoter, known as TR-L1p or ED-L1Ep, is activated independently of EBNA2 via STAT3, Sp1 and Sp3 transcription factors [52–54]. However, the EBNA2-dependent expression of LMP1 in B cells and the EBNA2-independent expression in epithelial cells cannot be explained simply by the use of different promoters, since the proximal ED-L1p can be also activated in epithelial cells, and LMP1-induced signalling pathways have been shown to auto-activate LMP1 transcription from the ED-L1p independently of EBNA2 [55,56]. It should also be noted that the levels of LMP1 and LMP2 in Latency II type infection can be quite variable even within a type of malignancy. For example, whilst LMP2A transcripts are consistently expressed in NPC tumours, LMP1 transcripts are detected in only a proportion of cases [38,39,57]. In this context, it may be relevant that LMP2A expression has been reported to inhibit LMP1 transcription in epithelial cells [58].

### 2.3 Other types of latent infection in BL

At least two other types of latent gene expression are associated with BL. One, which involves expression of all six EBNAs but not LMP1 or LMP2, is probably very rare and may be a consequence of integration of the viral genome into the cellular chromosome [59]. Another type of latent infection, in which all of the EBNAs except EBNA2 are expressed in the absence of LMP1 or LMP2 (Fig. 1), is more common and may be exhibited by more than 15% of endemic BL cases [25,26,59]. This latter type of latency arises because the tumours carry an episomal viral genome with a deleted sequence that removes the EBNA2 gene. For reasons that are not yet understood, the Wp promoter rather than the Qp promoter is activated for expression of EBNA transcripts, hence they are designated ‘Wp-restricted latency’ BLs. Most importantly, the Wp-restricted latency BL cells express BHRF1, a viral protein not previously associated with latency [27]. This homologue of cellular Bcl-2 [60,61], was previously regarded as being a lytic cycle gene product but is regularly expressed in Wp latency and confers a survival advantage to the tumour cells [27]. EBNA2-deleted viruses can arise, albeit rarely, in cancer-free people [62,63] but since they cannot express essential transforming genes, these deleted viruses cannot replicate by growth-transforming normal B cells and cannot therefore efficiently colonize the normal B cell pool. That Wp-restricted latency BLs are comparatively common amongst endemic BL tumours, suggests that there must be some selection advantage during the pathogenesis of BL; this is despite the expression of immunodominant target antigens for CD8^+^ immune T cell responses such as EBNA3A, EBNA3B and EBNA3C. We shall return to this issue later in the review.

Some BL tumours will successfully adapt to tissue culture and establish as cell lines. During early passages, EBV-positive BL lines typically display the same pattern of EBV latency as the original tumour *in vivo*, but during longer-term culture many lines drift towards Latency III [18,23]. Concomitant with the broadening of EBV latent gene expression, BL lines acquire a more differentiated phenotype with a loss of germinal centre markers, such as CD10 and Bcl-6, and an upregulation of activation markers, such as CD40 and various adhesion molecules [18,23,64]. The drift of BL lines towards Latency III is unlikely to be just an *in vitro* phenomenon, as immunohistochemical staining of some BL biopsies suggest that isolated cells within a predominantly Latency I expressing tumour may in fact express LMP1 and EBNA2 [65]. Why these Latency III cells do not go on to dominate the tumour mass, could be a result of the decreased rate of proliferation and the increased immunogenicity of these cells [23,66,67].

The BARF1 protein is a second putative oncoprotein of EBV [68,69]. Originally, BARF1 was thought to be exclusively an early lytic cycle product, although there is now evidence that it can be expressed during latency (e.g. [70,71]). BARF1 expression has been detected in BL biopsies [70,72], NPC [73], and gastric cell carcinoma [74]. In some of these studies it cannot be excluded that that BARF1 expression is due to a small number of cells in lytic cycle, but whether or not it is expressed in latency or in a small subpopulation of cells in lytic cycle, BARF1 could still contribute to the malignant phenotype since it is secreted as a soluble molecule.

### 2.4 Limitations of Latency nomenclature

The Latency I, II, III nomenclature was originally defined on the basis of the pattern of EBNA and LMP protein expression in BL and EBV-transformed normal B cell lines [24]. Since EBNA1 is essential for replication and maintenance of the viral episome, the most restricted form of latency possible in this system is Latency I. In non-dividing cells, however, there is no longer a strict requirement for EBNA1 expression. Consequently, a ‘Latency 0’ in which no viral antigens are expressed is an additional option for asymptomatic EBV persistence in healthy carriers.

An alternative nomenclature was recently suggested which has the merit of simplifying EBV gene-expression patterns and fitting them into an elegant hypothesis for how EBV persists in the healthy infected host [75]. In this alternative nomenclature, the terms “growth program”, “default program” and “latency program” roughly correspond to Latency III, Latency II, and Latency 0 (no EBV protein expression), respectively. However, when the alternative nomenclature is applied to EBV-induced malignancies, it is inadequate to cope with the complexity of EBV gene expression, a situation that is exacerbated further as new patterns of EBV gene expression are identified.

Whichever nomenclature is used, care must be taken to recognize their limitations. It would be inappropriate, for example, to classify a tumour as Latency III when immunohistochemistry showed that all the malignant cells were EBNA1^+^ but only rare cells were also EBNA2^+^ and LMP1^+^. Analysis of the same tumour sample by immuno-blot or by RT-PCR is inherently more prone to misleading interpretations. Nevertheless, quantitative RT-PCR for the complete range of latent genes in the tumour, and comparison with expression in standard control cell lines, coupled with careful analysis of Cp/Wp/Qp promoter usage, will usually indicate the correct interpretation [76].

### 2.5 Non-coding RNA transcripts

**Fig. 2:**
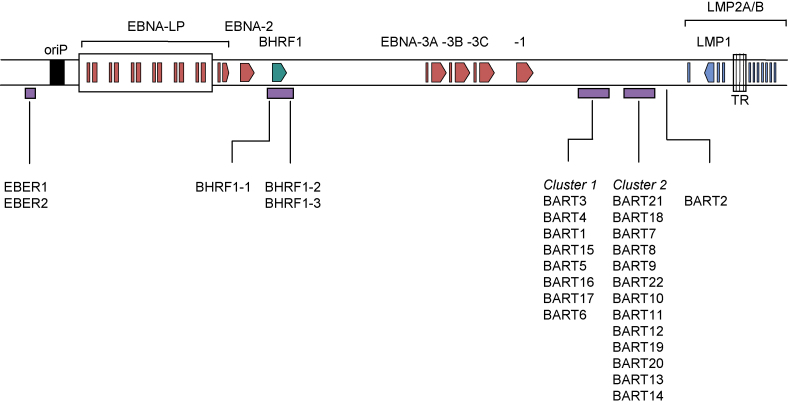
Expression of non-coding EBV RNAs. Schematic illustration of the EBV genome showing the location of the non-coding EBER RNAs, BHRF1 miRNAs and BART miRNAs. The non-coding nuclear EBER1 and EBER2 RNAs are transcribed by RNA polymerase III and are the most abundant viral transcripts in latently infected cells. The BamHI A region gives rise to a family of highly spliced BamHI A rightward transcripts (BARTs) which are also expressed in all forms of latency. While the protein-coding function of these BART RNAs remains controversial, at least 21 miRNAs are generated from BART RNA-derived introns. A second set of miRNAs, derived from the BamHI H region, are thought to be generated from Wp/Cp-initiated transcripts. Also shown are the location of the latent cycle proteins, latent origin of replication (oriP) and terminal repeats (TR).

In addition to protein-coding transcripts, EBV generates abundant non-coding transcripts (Fig. 2). The best studied are the RNA polymerase III transcribed small non-polyadenylated RNAs, termed EBER1 and EBER2, which are the most abundant viral transcripts in latently infected cells [77–79]. Although it has been reported that some lytically infected cells may not express EBERs [80], they are abundantly expressed in all types of latent infection. Indeed, the levels of EBERs are such that their detection by *in situ* hybridisation is considered to be one of the most reliable indicators of latent EBV infection at the single cell level in normal and diseased tissues.

A complex cluster of non-polyadenylated RNAs map to the BamHI A region [40,81], and are collectively known as BARTs (BamHI A rightward transcripts). It is not clear whether these rightward transcripts encode protein [82], but they do give rise to several micro-RNA (miRNA) species. Mature miRNAs are small (about 21–24 nucleotides) single-stranded RNAs processed from stem-loop containing double-stranded RNA precursors, and they regulate gene expression by binding to complementary sequences in the 3′ untranslated regions of target mRNAs to inhibit their translation or to direct their degradation [83]. It was first reported in 2004 that EBV encodes miRNAs [29]. They map to two regions of the genome: the BamHI H region (EBV-miR-BHRF1s in 5′ and 3′ sequences flanking the BHRF1 open reading frame), and the BamHI A region (EBV-miR-BARTs, processed from the introns of the BARTs). The virus encodes at least 3 EBV-miR-BHRF1 and 21 miR-BART miRNAs [29–31,84]. Additional low-abundant species exist, mostly arising from less efficient processing of the complementary 5′ or 3′ arm of the short hairpin pre-miRNAs.

The BARTs are transcribed across a region of the viral genome which includes an 11.8 kbp sequence that is deleted in the prototype B95.8 EBV [85], thereby limiting B95.8 EBV to just 5 (miR-BART-1, -2, -3, -4, 15) of the 21 most abundant miRNAs from this region of the genome. The miR-BARTs are expressed in all forms of latency and in lytic cycle. Some studies suggest that miR-BARTs are more highly expressed in epithelial cells than in B cells, and that in B cells the miR-BARTs may be more highly expressed in Latency III than in Latency I (e.g. [30,86–88]). However, accurate interpretation of these data is difficult because assays with different degrees of sensitivity and quantitation have been used in different studies, and mostly with a small number of long-established cell lines.

In contrast to the miR-BARTs, the BamHI H-derived miRNAs (miR-BHRF1-1, 1-2 and 1-3) show a more restricted expression pattern. BHRF1 transcripts in latent infection are mostly, if not exclusively, activated from the Cp/Wp promoters [29,30] and, therefore, no miR-BHRF1s are detected in latently infected epithelial tumours [88]. As for the miR-BARTs, the available evidence from quantitative analysis of miR-BHRF1 expression in different types of latency in B cell lines (e.g. [30,84,86]) does not yet allow conclusive inferences to be made with regards to a possible correlation between the type of EBV latency and the expression of miR-BHRF1s. In lytic cycle, BHRF1 transcripts initiate from a promoter proximal to the BHRF1 open reading frame, and overlapping the miR-BHRF1-1 sequence, so that only miR-BHRF1-2 and miR-BHRF1-3 can be processed [30].

## 3 EBV gene expression and the pathogenesis of BL

The concept, that the ability of EBV to growth-transform normal B cells is directly responsible for the pathogenesis of BL, was weakened by the demonstration that BL tumours have a more restricted pattern of gene expression than EBV-transformed LCLs. The validity of the concept was further questioned following a series of studies with recombinant EBV lacking expression of individual latent genes, which revealed that the loss of expression of any one of EBNA1, EBNA2, EBNA3A, EBNA3C or LMP1 was sufficient to substantially or completely abrogate the ability to generate LCLs following infection of primary B cells in culture (reviewed in [89,90]).

It is generally accepted that deregulated expression of the c-myc proto-oncogene, resulting from the chromosomal translocations that are characteristic of all BLs, is the primary oncogenic event in the pathogenesis of BL irrespective of the association with EBV. What, then, is the role of EBV? There are two broad (and not mutually exclusive) hypotheses: (i) EBV might increase the likelihood of genetic accidents giving rise to the translocation, and (ii) EBV might complement the activity of c-myc. The fact that BL tumours have phenotypic characteristics of germinal centre cells [91] provides one clue to the pathogenesis of BL that is supported by what is known about two other co-factors for BL; malaria and HIV.

In areas of endemic BL, an important co-factor with EBV for lymphomagenesis is *P. falciparum* malaria (reviewed in [92]). Two features of malarial infection likely to be relevant to BL are an immunosuppressive effect on EBV-specific T cell immunity [93–96], and a mitogenic effect on B cells [97,98]. The immunosuppressive effect may be responsible for the fact that children in areas of holoendemic malaria have very high loads of EBV in the peripheral blood, and these levels are further elevated during acute malarial infection [99–101]. The mitogenic effect of *P. falciparum* malaria on B cells, leading to increased germinal centre activity, is also likely to be critical since BL displays many markers of germinal centre cells and the c-myc/Ig gene translocations are likely to be accidents of immunoglobulin gene rearrangements in the germinal centre. In normal circumstances, cells carrying illicit immunoglobulin gene recombinations would be programmed to self-destruct, but the elevated EBV loads in malarial patients would increase the likelihood of such cells being infected with EBV and rescued from cell death. Another major co-factor for BL, is HIV. In contrast to malaria-associated BL, AIDS-associated BL arises in patients who remain relatively immunocompetent [102]. HIV patients do not show the same high EBV loads that are found during malarial infection, and only 30–40% of AIDS-associated BL tumours are EBV-positive. Similarly to malaria, however, HIV infection also activates the B cell system [103]. Together, these observations are consistent with a mechanism where increased germinal centre activity is the critical factor for the generation of c-myc translocations. The increased EBV load associated with malarial infections increases the likelihood that such cells will be rescued from the pro-apoptotic effects of activated c-myc by infection with EBV.

That EBV complements the functions of c-myc in BL, is consistent with experimental evidence (see below). Furthermore, if an activated c-myc is experimentally over-expressed in EBV-transformed LCLs, then proliferation of the cells becomes independent of EBNA2 and LMP1. In addition, inhibition of EBNA2 function and, therefore, LMP1 expression causes the LCLs with activated c-myc to assume many of the phenotypic characteristics of BL lines [104,105]. Indeed, expression of the full Latency III set of EBV genes, particularly EBNA2 and LMP1, appears to be incompatible with activated c-myc [105]. On the other hand, one or more of the restricted set of genes expressed in BL appears to be able to complement activated c-myc in BL.

Over-expression of c-myc is a common feature of many cancers. It is a transcription factor that can bind to around 15% of promoters on the human genome [106]. Consequently it influences many facets of cell behaviour, including proliferation and apoptosis (reviewed in [107]). In normal cells, c-myc expression is tightly regulated and its functions that promote proliferation (including upregulation of cyclin D and E, and downregulation of p27) are counterbalanced by c-myc-induced apoptotic checkpoints which include the ARF/MDM2/p53 and RB pathways. Genes involved in the p53 pathway are frequently mutated in BL (reviewed in [108]). More recently, animal models of c-myc induced tumours have implicated the pro-apoptotic BH3-only protein (Bim) as a p53-independent apoptotic checkpoint [109,110].

Against this background, it is tempting to speculate that EBV might complement c-myc in BL by ablating the apoptotic activity of the oncoprotein. Exactly how the EBV genes might effect this complementation is currently under investigation in a number of laboratories, but one important clue is provided by observations on Bim, which is downregulated in normal B cells following infection and growth-transformation with EBV *in vitro*[111]. Bim is highly expressed in Latency I BL but is substantially downregulated in Wp-restricted BLs [27,112]. This suggests that downregulation of Bim might be a complementing factor in the Wp-restricted subset of BLs. Although downregulation of Bim is not seen in Latency I BLs, and therefore is not likely to be involved in most cases of BL, the observation with Wp-restricted BLs does lend credence to the idea that the pro-apoptotic checkpoints which regulate c-myc-induced proliferation in normal cells are targeted by secondary genetic changes and/or by EBV genes in the pathogenesis of BL.

### 3.1 EBV promotes survival and tumourigenicity in BL lines

What, then is the evidence that EBV might regulate apoptosis pathways in BL? An important tool for answering this question was provided by the fortuitous discovery that the maintenance of the viral episome in some BL lines is unstable, so that it is possible to establish rare EBV-loss subclones in culture. EBV-positive lines in Latency I show a small but reproducible survival advantage over EBV-loss subclones when subjected to apoptotic triggers [59,113,114]. The mechanism of this protection is not yet known, even though the number of EBV genes potentially involved is very limited. Interestingly, EBV-positive BL lines displaying a Wp-restricted form of latency show a substantially enhanced protection from apoptotic triggers [27,59,112].

### 3.2 Contribution of EBNA1 to BL

EBNA1 is expressed in all EBV-positive BLs. It is essential for the replication of the EBV episome in proliferating cells [115,116]. In addition, the glycine-alanine repeat sequence of EBNA1 limits its own translation efficiency and to some extent modulates antigen processing to impair its own recognition by EBNA1-specific CD8^+^ T cells [117,118]. While the episomal maintenance and immune-modulating functions of EBNA1 are both relevant to BL pathogenesis, they cannot themselves account for the enhanced survival and tumourigenicity of EBV-positive BL lines relative to rare subclones that have lost the EBV genome in culture. Nevertheless, EBNA1 appears to have additional functions. Thus, expression of a dominant-negative mutant molecule in EBV-transformed LCLs and in BL cells, to specifically inhibit the transcriptional functions of EBNA1 while retaining the episome maintenance function, resulted in a dose-dependent decrease in cell survival [119]. Conversely, expression of EBNA1 in some EBV-negative lines was shown to inhibit p53-dependent apoptosis [119].

### 3.3 Contribution of BARF1 to BL

BARF1 has been shown to enhance cell survival, clonability, and tumourigenicity of EBV-loss subclones of the Akata BL line [120]. However, while the parental EBV-positive Akata cells expressed abundant BARF1 transcripts, immunostaining for BARF1 protein showed that it was actually expressed in only a small subpopulation of cells; i.e. cells in lytic cycle. Furthermore, the anti-apoptotic effect of BARF1 appears to be mediated via induced expression of Bcl-2, which is not expressed in the parental EBV-positive Akata cells nor in other Latency I BLs.

### 3.4 Contribution of EBERs to BL

The EBERs have been reported to restore a tumourigenic phenotype to BL cells that have lost the EBV genome in culture [121–123]. Through interaction with RIG-1 (retinoic acid-inducible gene-1), the EBERs can induce anti-inflammatory cytokines, such as IL10 [124] and type I interferon production [125]. They may also confer resistance to interferon-α induced apoptosis through binding to PKR (RNA-activated protein kinase) and inhibiting its phosphorylation [126,127]. The contribution of EBERs to the tumourigenicity of BL lines is independent of the inhibition of PKR [122] but might be related to induced expression of Bcl-2 [121]. It has been suggested that EBERs induce expression of IL-10, which can act as an autocrine growth factor to enhance survival of BL cells [128]. The generality of some of these observations is questioned by other studies: Bcl-2 is not normally expressed in BL tumours [129]; and cells infected with EBER-negative recombinant EBV or wild-type EBV did not differ in their sensitivity to interferon-mediated inhibition of proliferation nor the inhibitory effect of interferon on vesicular stomatitis virus replication [130]. Nevertheless, recombinant EBV lacking EBERs is less efficient at transforming normal B cells [131]. In conclusion, whilst the EBERs undoubtedly have the potential to influence cellular phenotype and immune-responses, the molecular mechanisms and their exact roles in the normal life-cycle of EBV and in the pathogenesis of EBV-associated malignancies remain unclear.

### 3.5 Contribution of miRNAs to BL

The functions of some EBV miRNAs are beginning to be identified, and their roles in EBV-associated malignancies may soon be become apparent. The miR-BARTs are regularly expressed in BLs, albeit often at lower levels than in EBV-associated epithelial tumours. It should be noted, however, that the BARTs are not essential for EBV transformation of normal B cells to LCLs [132], and that a deletion in the prototype B95.8 strain of EBV removes most of the miR-BARTs [30] while retaining transformation function. In addition, the miR-BHRF1s may not be regularly expressed in BL tumours. Nevertheless, there remains the possibility that specific EBV miRNAs might enhance certain functions of EBV or c-myc in BL, as EBV miRNAs have been shown to modulate expression of other EBV genes [133,134] as well as cellular genes [135,136]. Regarding the regulation of cellular genes by EBV miRNAs, the effect of miR-BART5 on PUMA (the p53 Up-regulated Modulator of Apoptosis) is of immediate interest since PUMA regulates apoptosis via p53 dependent and independent mechanisms [136]. However, even in NPC where miR-BART5 is highly expressed [88,136], 40% of tumours do not show a reduction in PUMA expression [136], suggesting that an effect of miR-BART5 on cell survival through regulation of PUMA is not a consistent feature of this NPC. Evidence for a role for EBV-miRNAs in BL has yet to be reported.

### 3.6 Contribution of BHRF1 and EBNA3 proteins to BL

The identification of a subset of endemic BLs which carry EBV in a Wp-restricted latency, and are concomitantly strongly resistant to apoptotic triggers, adds further weight to the idea that the role of EBV in BL is to counteract the pro-apoptotic influence of deregulated c-myc [25]. While a Latency I infection appears to provide low level protection from apoptotic triggers, the broadening of viral antigen expression in Wp-restricted latency BLs to include the viral Bcl2 homolog, BHRF1, and the EBNA3 family of proteins is associated with much stronger protection from apoptosis and a complementation of c-myc induced lymphomagenesis [27,59,112].

The detection of BHRF1 expression in Wp-restricted BL cells, resulting from the close proximity of the BHRF1 gene to the highly active BamHI W promoter as a consequence of the intervening EBNA2-deleted sequences, provided a clear explanation for their survival advantage. BHRF1 is a Bcl2 homologue known to be capable of protecting B cells from growth factor withdrawal, TRAIL death receptor signalling, irradiation and chemotherapeutic drugs [61,137,138]. Until recently, however, BHRF1 expression was thought to be restricted to the lytic replication cycle of EBV, and had not been detected in malignant cells. There is now growing evidence that the two viral Bcl2 homologues encoded by EBV (BHRF1 and BALF1) play important roles not only in lytic cycle, but also in latently infected cells where they contribute to the initial stages of growth transformation [139] and possibly also, in the case of BHRF1, to the continued growth or survival of EBV-transformed cells [27]. In the context of BL, it was shown that ectopic expression of low levels of BHRF1 in EBV-negative and Latency I BLs was sufficient to confer almost complete protection from ionomycin and anti-IgM induced apoptosis, suggesting that the natural expression of BHRF1 in Wp-restricted BLs contributes to the enhanced resistance of this subset of BLs to these apoptotic triggers [27].

A role for EBNA3A and EBNA3C together in apoptosis protection has also been reported and has implications for Wp-restricted BL cells [140]. Here, EBV-negative BL cells were infected with recombinant EBV genomes and, using drug selection, stable converted cell lines were established. Although the BL phenotype was not fully recapitulated since infection with recombinant EBV genomes results in a Latency III-like infection with expression of antigens not normally expressed in BL cells, such as EBNA2 and LMP1, it was noted that BL cells infected with either EBNA3A-deleted or EBNA3C-deleted recombinant EBVs were sensitized to death induced with nocodazole, cisplatin or roscovitine [140]. This suggests that EBNA3A and EBNA3C can act co-operatively to protect BL cells from these apoptotic triggers, and it reveals a second mechanism through which EBV can complement c-myc actions in BL lymphomagenesis. The relative contributions of BHRF1 and EBNA3A/EBNA3C to Wp-restricted BL pathogenesis will become clearer with detailed investigations into their mechanisms of action, including the apoptotic pathways they influence. Further investigations will also reveal to what extent BHRF1 and EBNA3A/EBNA3C co-operate with, or mediate their effects through, downregulation of Bim.

## 4 Immune modulating mechanisms in BL

T cell immune responses play an important role in preventing the outgrowth of EBV-transformed B cells in healthy infected individuals (reviewed in [4]), but they are clearly ineffective at preventing tumour growth in BL patients. To some extent, this may be due to the immunosuppressive effects of *P. falciparum* malarial infections in regions where BL is endemic [93,94,96], although the potency of some EBV-specific EBV responses is restored following recovery from an acute malarial attack [94]. More importantly, perhaps, BL tumour cells display an inherent resistance to EBV-specific CD8^+^ immune effectors [17,141,142].

The identification of restricted Latency I EBV expression in BL tumours offered a simple explanation for the resistance to CD8^+^ T cell responses. The repression of immunodominant viral target antigens [143,144] that are expressed in LCLs, and the partial protection offered to EBNA1 by its glycine-alanine domain [117,118,145] would surely enable these tumours to evade immune responses? More recent studies have questioned the extent to which the processing defect of EBNA1 might render this protein immunologically silent [146–148]. Furthermore, Wp-restricted BLs (which do express the immunodominant EBNA3A, 3B, 3C family of latent genes) are also poorly recognized by CD8^+^ immune effectors [25]. This latter result in particular highlights the fact that BL cells have a more general impairment of antigen presenting function irrespective of EBV status. Notably, BL cells express reduced levels of adhesion molecules [64] that are required for efficient conjugate formation with effector T cells, and of HLA class I molecules [19,141,142] which present antigenic peptides at the cell surface for recognition by the T cell receptor of CD8^+^ effector T cells. In addition to a general reduction in cell surface HLA class I molecules, certain HLA alleles are selectively downregulated to a greater degree [149]. BL cells also show reduced levels of the TAP1/TAP2 transporter complex [67,150] which mediates transport of antigenic peptides from the cytosol to the ER where they associate with MHC class I molecules. Together, these features of the BL cell phenotype render the tumour cells resistant to CD8^+^ effector T cell recognition even when appropriate endogenous target antigens are expressed. This resistance is reversible following expression of LMP1, which upregulates expression of the adhesion molecules, TAP1/TAP2 transporters and HLA class I molecules [67,151,152].

The effect of the BL tumour phenotype on HLA class II antigen presentation is less well studied. Early work showed that the ability of BLs to process antigen via HLA class II and stimulate CD4^+^ T cell responses was impaired in BL tumour lines [153]. More recent work, however, shows that BL cells can be killed by EBNA1-specific CD4^+^ effector T cells [154], and Wp-restricted BLs can present EBNA3C epitopes to CD4^+^ T cells [155]. Such CD4^+^ immune responses to EBNA1 appear to be relatively common in healthy infected individuals, and an animal model demonstrated that these immune responses are potentially able to prevent the growth of BL tumours *in vivo*[156]. Interestingly, however, EBNA1-specific CD4^+^ responses are selectively impaired in children with endemic BL [157].

## 5 Lessons from BL for the biology of EBV

BL was the first well-documented example of a human tumour showing a consistent impairment of antigen presenting function [19,67,142,150] and has become a paradigm for immune-escape by tumour cells of both viral and non-viral associated cancers. There is also the possibility that defects in HLA class I antigen presentation in BL and other cancers might directly enhance tumour growth by affording protection from apoptotic stimuli [158].

One important lesson from studies on BL lymphoma was that neither the viral gene expression nor the cellular phenotype is a unique feature of malignant cells. This was speculated before restricted forms of latency were identified in long-term healthy carriers [159–162], and before the cell surface marker expression of BL tumour cells was shown to have a normal counterpart in the germinal centre [91].

**Fig. 3:**
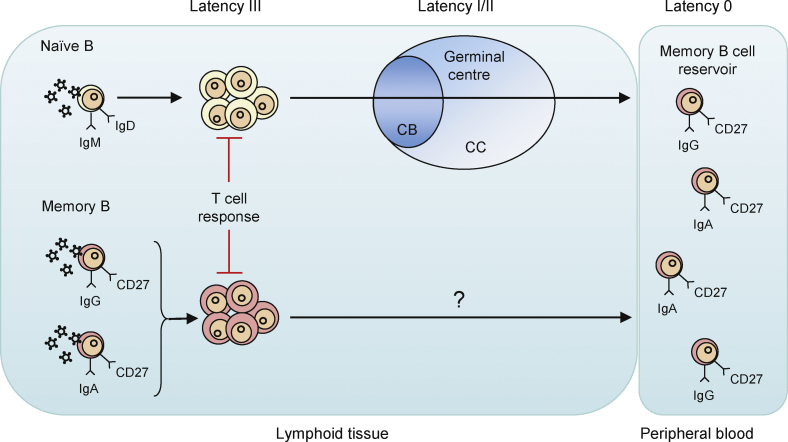
Selective colonization of the memory B cell compartment during primary EBV infection. How EBV achieves selective colonization of the isotype class-switched memory B cell pool *in vivo* remains unclear. One hypothesis [75] proposes that incoming virions preferentially infect IgM^+^IgD^+^ naïve B cells which, as a result of a transient growth-transforming infection mimicking antigen stimulation, are driven to form a germinal centre (GC). Thereafter the physiologic processes of somatic hypermutation and class switch recombination come into play and deliver latently infected IgG^+^CD27^+^ or IgA^+^CD27^+^ GC progeny cells into the long-lived memory compartment. An alternative hypothesis, based on studies of individual cells micro-dissected from infectious mononucleosis lymphoid tissues [184], questions the involvement of GC transit and instead envisages the preferential infection and/or survival of memory cells post-infection, compared to their naïve counterparts. These models are not mutually exclusive, and both share one key feature; that downregulation of viral antigen expression is linked to the normal differentiation of B cells.

This lesson, together with the observation that the virus:cell interaction in BL was effectively immunologically silent for CD8^+^ T cell responses (see previous section), provided the foundation for our current understanding of how EBV normally colonizes the B cell compartment and persists in healthy carriers (Fig. 3). Thorley-Lawson's group has been particularly active in elucidating the mechanisms of EBV persistence, and one of their key observations was that EBV-infected cells in the blood of long-term carriers are confined to the memory B cell subset and are absent from the larger, naïve B cell subset [163,164]. This led to the development of a model for EBV colonization of the B cell pool which reflects the normal physiological process of normal B cell development [75] and is broadly supported by a large body of evidence from the Thorley-Lawson group and others. The fact that LMP1 can reverse the antigen presentation defect in BL tumour cells [67] suggests that this viral oncoprotein provides an immunological check to counter the uncontrolled growth of LCL-like cells in immunocompetent infected individuals. In long-lived memory B cells carrying EBV as a latent infection *in vivo*, the cells do not need to proliferate and so they can support the more restricted types of latency where critical transformation-associated latent genes are not expressed [162], and where there is no need for LMP1 to enhance immunogenicity.

A corollary of the hypothesis for EBV persistence in normal carriers is that the stage of B cell development determines EBV gene expression and that mechanisms must exist for switching infected cells between different forms of latency. There is undoubtedly a correlation between EBV gene expression and cellular gene expression both in normal and in malignant cells, although most studies have used experimental models that demonstrate EBV genes driving the cellular phenotype, rather than *vice versa*. Thus, transfection of individual EBV latent genes, particularly LMP1 and EBNA2 into primary B cells or EBV-negative or Latency I BL cells drives the cellular phenotype towards a more differentiated activated lymphoblast phenotype (reviewed in [90]). Reversal of the lymphoblastoid phenotype in a conditional LCL model to a germinal centre phenotype can be achieved by inhibiting the function of EBNA2, and thus also the expression of LMP1, provided that activated c-myc is constitutively expressed [105]. There is, however, also evidence to support the view that the cellular phenotype can determine EBV gene expression. For example, activation of the Wp and Cp promoters immediately following infection of resting B cells *in vitro* is dependent upon expression of the BSAP/PAX-5 transcription factor whose expression is B cell specific [46], and the silencing of latent gene promoters can occur by epigenetic mechanisms mediated by cellular genes [165–171]. Furthermore, infection of chronic lymphocytic leukaemia B cells with EBV *in vitro* results in an unusual type of latent gene expression where the EBNAs are expressed in the absence of LMPs and the cells are not growth-transformed [172,173], whereas prolymphocytic leukaemia B cells express the full Latency III spectrum of EBV genes and are transformed [173]. Infection of an EBV-negative Hodgkin's lymphoma cell line (KMH2), which has germinal centre characteristics, results in a Latency I or Latency II type of EBV gene expression [174,175]. The mechanisms by which the differentiation state of the host B cell determines viral gene expression remain to be elucidated, but this information is critical for a more complete understanding of the normal carrier state.

The impairment of HLA class I antigen presentation in BL provides an explanation for how BL tumours grow in the face of relatively intact T cell responses, and the enhancement of antigen presentation in Latency III type infection might explain why immunocompetent long-term carriers of EBV do not usually succumb to B cell lymphoproliferations arising from growth-transformation of normal B cells. Until recently, the enhanced antigen presentation of normal primary B cells following infection *in vitro* suggested that EBV was somewhat distinct from other herpesviruses which encoded genes that directly interfered with antigen presentation. However, the immune-modulating genes of other herpes viruses are generally expressed during lytic cycle, and we now know that EBV similarly encodes immune evasion genes during its own lytic cycle. Thus, immune recognition of EBV target antigens during lytic cycle is impaired through BGLF5, BNLF2a, BILF1 (vGPCR), and BCRF1 (vIL10) acting at different points of the HLA class I antigen presentation pathway, and BZLF1, BZLF2 and BGLF5 acting on the HLA class II antigen presentation pathway (reviewed in [176]). This highlights a point that although lytic cycle gene products are the most frequent targets for EBV-specific CD8^+^ immune-T cells in healthy carriers, it does not necessarily follow that those responses effectively eliminate infected cells that have entered lytic cycle. In fact, NK cells might be more important for preventing the excessive production of virus progeny in primary infection and healthy carriers [177,178].

Another key lesson from BL was that protection from apoptosis, which is important for the growth of this tumour, can be induced by EBV latent genes [179]. The first EBV gene that was identified as having anti-apoptotic properties was LMP1, which acts by inducing the anti-apoptotic proteins, Bcl-2, Mcl-1, and bfl-1 [129,180,181]. To varying extents, EBNA2 [182], LMP2A [183] and EBNA3A/C [140] can also enhance cell survival. These latent gene products are all essential for the ability of EBV to growth-transform primary B cells. This indicates that EBV-induced growth transformation is not simply a matter of triggering proliferation by activating cell cycle, but critically it also involves modulation of apoptosis pathways to enhance cell survival. With regards to BL, the latent genes LMP1, LMP2A, and EBNA2 are not normally expressed in the tumour cells, yet EBV does confer a survival advantage in Latency I BLs; particularly so in Wp-restricted Latency BLs. The study of Wp-restricted BLs has highlighted an important role for BHRF1, both as a contributor to the pathogenesis of a subset of BL tumours and also as a gene involved in the establishment of *in vitro* transformed LCLs. Altmann and Hammerschmidt showed that immediate inhibition of apoptosis is an essential requirement following *in vitro* infection of primary B cells and that the two viral homologues of Bcl-2, BHRF1 and BALF1, were transiently expressed during the early stages of transformation [139]. This remarkable result was unexpected since both BHRF1 and BALF1 were considered to be lytic cycle genes. Their expression was attributed to promiscuous expression of the unmethylated EBV genome shortly after viral entry. However, more recent results [27] show that latent BHRF1 transcripts are driven from the Wp-promoter so that this protein is co-expressed with EBNA-LP and EBNA2 shortly after infection. Furthermore, low levels of BHRF1 protein continue to be expressed in some established LCLs where some Wp promoter activity is retained.

From the examples illustrated here, it is clear that the study of BL has provided us with invaluable clues to the biology of EBV in asymptomatic carriers and to its association with malignant disease. EBV and BL have together provided paradigms for understanding the intricate interactions of viruses and tumours with the immune system. Fascinatingly, this well-worn model continues to surprise us with new details that have important implications for EBV and the wider field of viral oncology.

## Conflict of interest statement

None.
